# Severe Decompression Sickness Associated with Shock and Acute Respiratory Failure

**DOI:** 10.1155/2020/8855060

**Published:** 2020-11-03

**Authors:** Abdullah Arjomand, James R. Holm, Anthony J. Gerbino

**Affiliations:** ^1^Sections of Graduate Medical Education, Virginia Mason Medical Center, Seattle, WA, USA; ^2^Undersea and Hyperbaric Medicine, Virginia Mason Medical Center, Seattle, WA, USA; ^3^Pulmonary Medicine, Virginia Mason Medical Center, Seattle, WA, USA; ^4^Critical Care Medicine, Virginia Mason Medical Center, Seattle, WA, USA

## Abstract

Decompression sickness (DCS) is a well-recognized complication of diving but rarely results in shock or respiratory failure. We report a case of severe DCS in a diver associated with shock and respiratory failure requiring mechanical ventilation. A healthy 50-year-old male diver dove to a depth of 218 feet for 43 minutes while breathing air but omitted 6.5 hours of air decompression due to diver error. The clinical presentation was remarkable for loss of consciousness, hypotension, cutis marmorata, peripheral edema, and severe hypoxia requiring mechanical ventilation with diffuse lung opacities on chest radiograph. Laboratories were significant for polycythemia and hypoalbuminemia. A single hyperbaric oxygen treatment was provided on the day of admission during which shock worsened requiring aggressive volume resuscitation and three vasopressors. In the first 37 hours of hospitalization, 22 liters of crystalloid and multiple albumin boluses were administered for refractory hypotension by which time all vasopressors had been discontinued and blood pressure had normalized. He required 10 days of mechanical ventilation and was discharged on day 21 with mild DCS-related neurologic deficits. This clinical course is characteristic of DCS-related shock wherein bubble-endothelial interactions cause a transient capillary leak syndrome associated with plasma extravasation, hemoconcentration, and hypovolemia. The pathophysiology and typical clinical course of DCS-related shock suggest the need for aggressive but time-limited administration of crystalloid and albumin. Because hyperbaric oxygen is the primary treatment for DCS, treatment with hyperbaric oxygen should be strongly considered even in the face of extreme critical illness.

## 1. Introduction

Decompression sickness (DCS) is a well-recognized complication of diving that occurs when inert gas breathed at depth leaves solution and forms injurious bubbles. Symptoms are most often musculoskeletal and neurologic and are effectively treated with hyperbaric oxygen using a standard protocol administered over roughly five to eight hours.

Shock is a rare manifestation of DCS that is typically associated with a large, omitted decompression obligation. These patients often present with severe illness, challenging the clinical team to provide aggressive critical care that transitions from the emergency department, to the hyperbaric chamber, and then to the intensive care unit. We report a case of DCS causing severe shock associated with plasma extravasation, hemoconcentration, and respiratory failure requiring mechanical ventilation.

## 2. Case

A healthy 50-year-old experienced SCUBA diver dove to a maximum depth of 218 feet for 43 minutes while breathing air. He became confused due to nitrogen narcosis leading to a longer, deeper dive than originally planned. He ascended from depth slowly according to the staged decompression suggested by his dive computer but ultimately exhausted his supply of breathing gas and was forced to surface having omitted 6.5 hours of required air decompression. Upon surfacing, he was conscious and aware that he had a massive unfulfilled decompression obligation, hailed a nearby boat, and requested that emergency medical services be summoned. Shortly thereafter, he lost consciousness but remained at the surface due to his buoyancy control device. He was rescued from the water, intubated, and mechanically ventilated.

**Figure 1:**
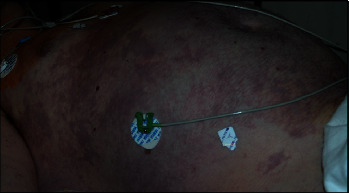
Cutis marmorata, a rash typically associated with severe decompression sickness, was present on arrival to the hyperbaric chamber.

Upon arrival in the emergency room, he was hypotensive requiring norepinephrine infusion. Peripheral edema was present. He developed cutis marmorata (Figure 1) [1], a rash typically associated with severe DCS. The initial chest radiograph (after 2 L intravenous crystalloid) demonstrated diffuse opacities. Initial labs showed hypoalbuminemia (1.1 g/dL), polycythemia (hematocrit 58), lactic acidosis, and impaired gas exchange (pH 7.18, P_a_O_2_ 120 mm Hg, P_a_CO_2_ 50 mm Hg on 100% oxygen).

He was transported to a hospital-based multiplace hyperbaric chamber with critical care capabilities. The standard hyperbaric treatment protocol for decompression sickness, a United States Navy Treatment Table 6, was begun. Shock worsened during hyperbaric treatment prompting repeated boluses of intravenous crystalloid and albumin and addition of vasopressin and epinephrine infusions to norepinephrine. Similarly, progressive hypoxia, acidemia, and ventilator dyssynchrony during hyperbaric treatment prompted increases in positive end-expiratory pressure and initiation of bicarbonate and cisatracurium infusions. Despite this management, shock and hypoxia worsened and the decision was made to terminate the hyperbaric treatment after 230 minutes of a planned 260-minute treatment. Subsequent hyperbaric treatments were not provided due to cardiopulmonary instability.

**Figure 2:**
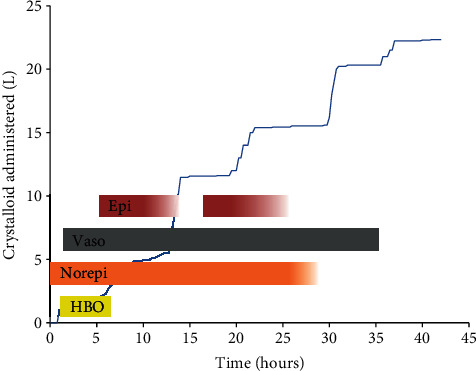
Time course of crystalloid administered upon arrival at the medical center with hyperbaric capabilities. Solid bars show timing of hyperbaric oxygen treatment (HBO) and norepinephrine (Norepi), vasopressin (Vaso), and epinephrine (Epi) infusions. The darkest color intensity indicates maximum vasopressor dose (Norepi, 30 mcg/min; Vaso, 0.04 units/min; Epi, 10 mcg/min), faded intensity indicates taper of dose, and absence of bar indicates discontinuation of vasopressor. Mean arterial pressure (not shown) was 55-65 mm Hg throughout this period.

During the initial 37 hours of hospitalization, 22 liters of crystalloid and multiple albumin boluses were administered for refractory hypotension at which time blood pressure normalized and all vasopressors had been discontinued (Figure 2). Despite aggressive diuresis over the next few days until euvolemic, he required low tidal volume mechanical ventilation for 10 days. He was discharged on day 21 without need for supplemental oxygen. Neurologic deficits included mild ischemic optic neuropathy and cognitive dysfunction with brain MRI demonstrating small, multifocal infarcts consistent with severe DCS.

## 3. Discussion

We present a case of severe DCS due to a massive omitted decompression requirement associated with shock, acute respiratory failure, and neurologic injury. This case highlights two rare manifestations of DCS—shock and respiratory failure—treated with hyperbaric oxygen therapy in the setting of cardiopulmonary collapse.

Only three cases of shock due to DCS have been reported in the last 45 years [2–4] but case series of DCS reported prior to that time in divers, aviators, and animal models reveal a characteristic clinical course. These accounts [5–8] describe hypovolemic shock with plasma extravasation associated with hemoconcentration and hypoalbuminemia, typically resolving within 48-72 hours if the subject survived. The rarity of these clinical reports and their publication outside of the critical care literature may make recognition of DCS-related shock challenging for the intensivist, especially when not associated with an exceptional dive profile.

The pathophysiology of shock due to DCS involves the interaction of undissolved inert gas with vascular endothelium. Inert gas breathed at high ambient pressure (i.e., at depth) forms bubbles in tissue and vascular spaces if, upon diver ascent, the pressure decreases too quickly relative to the amount of dissolved gas. In the present case, the omitted decompression was massive, resulting in an uncommonly large burden of bubbles. Bubble-endothelial interactions lead to endothelial dysfunction and an inflammatory response [9–12] that increases vascular permeability, resulting in plasma extravasation and subsequent intravascular volume depletion [7]. Whether there is a component of distributive shock related to endothelial dysfunction that compounds hypovolemic shock is unclear. This pathophysiology and characteristic clinical course suggest the need for aggressive, early but time-limited administration of crystalloid and albumin to correct intravascular hypovolemia and oncotic pressure defects.

We advocate for a trial of hyperbaric oxygen even in the most unstable of patients with DCS or arterial gas embolism because hyperbaric oxygen is the primary treatment for these diseases and rapid improvement in typical DCS-related symptoms is common [13]. Although shock did not improve during hyperbaric treatment in the present case, we speculate that clinical outcome would have been worse without such treatment.

There are several caveats to the aggressive use of hyperbaric oxygen in the critically ill DCS patient. The patient should be first transported to the closest emergency department for evaluation and stabilization, even if that center does not have hyperbaric capabilities. Second, the ability to comfortably manage severely ill patients is variable, even among hyperbaric centers with critical care capabilities [14, 15]. Finally, the decision to treat severely ill patients with hyperbaric oxygen requires close collaboration between the intensivist and hyperbaricist throughout the clinical course to repeatedly weigh the risks and benefits of treatment [14].

This patient's respiratory failure likely was multifactorial including water aspiration, lung DCS, extravascular fluid overload, and possibly DCS-related fat emboli. Water aspiration is likely because the patient lost consciousness at the surface prior to rescue. While hypotension predominates in DCS-related shock, varying degrees of lung injury are often reported [3, 5, 6] and interactions between pulmonary endothelium and bubbles result in vascular leak [10, 11]. DCS-related pulmonary edema is suggested in the present case by dense, homogeneous lung opacities on the admission chest radiograph (rather than patchy infiltrates anticipated from aspiration at the water's surface) and the massive systemic capillary leak that suggests similar injury to the pulmonary endothelium. Aggressive volume resuscitation undoubtedly contributed to respiratory failure, with effects likely amplified by increased permeability of the alveolar-capillary membrane. Although there is no evidence of fat emboli in the present case, fat emboli have been found in the lung and other organs in fatal cases of DCS [6, 8, 16], presumably due to bubble-mediated infarction of long bones, and thus may also play a role in DCS-related lung injury.

We have presented a case of DCS-related shock with acute respiratory failure requiring prolonged mechanical ventilation. This case illustrates the characteristic clinical course of time-limited shock with plasma extravasation caused by severe DCS. We recommend aggressive, early resuscitation with crystalloid and albumin to correct intravascular volume deficits and strong consideration of hyperbaric oxygen treatment even in extreme critical illness.

## Data Availability

Data are not applicable (all data related to the presented case is available in the medical record).
